# *Pseudomonas kielensis* str. Ze23jcel16 complete genome obtained through R10.4.1 Nanopore Flow cell chemistry

**DOI:** 10.1128/MRA.00714-23

**Published:** 2023-10-11

**Authors:** Lucas Serra Moncadas, Janis Rogenmoser, Cyrill Hofer, Angel Rain-Franco, Adrian-Stefan Andrei

**Affiliations:** 1 Microbial Evogenomics Lab, Limnological Station, Department of Plant and Microbial Biology, University of Zurich, Kilchberg, Zurich, Switzerland; 2 Faculty of Sciences, University of Zurich, Zurich, Zurich, Switzerland; 3 Limnological Station, Department of Plant and Microbial Biology, University of Zurich, Kilchberg, Zurich, Switzerland; The University of Arizona, Tucson, Arizona, USA

**Keywords:** genomics, *Pseudomonas*, DNA sequencing, long-read sequencing, cultivation

## Abstract

Here, we report the complete genome of *Pseudomonas kielensis* str. Ze23jcel16 isolated from a freshwater sample. The high-quality chromosome was obtained employing R10.4.1 Nanopore Flow cell chemistry and was assembled as a circular element at 45× coverage, a length of 5.8 Mbp, and a G+C content of 61.15%.

## ANNOUNCEMENT

*Pseudomonas* genus encompasses diverse species that colonize a plethora of environments ranging from soil to freshwater and sediments. Though renowned as potential pathogens of animals and plants, *Pseudomonas* species are ubiquitous copiotrophs capable of utilizing a wide range of carbon substrates (1). High-quality genomic data are required to be able to fully comprehend Pseudomonas’ diversity as well as lifestyle strategies.

The strain was isolated from surface waters of an alpine lake (Lake Zurich, 47°18′N, 8°34′E, Switzerland) by retrieving 500 mL of water with a Rutner sampler. Briefly, the strain was cultivated in agar plates (15 g/L) amended with modified artificial lake water (2) using glucose as a sole carbon source (100 µM final concentration). The strain was purified by sequentially streaking individual colonies and then cryopreserved as described elsewhere (3). Prior to DNA extraction using the Quick-DNA HMW MagBead kit (Zymo Research), the strain was grown in LB (Difco, 240210, 2 L). The obtained DNA was purified with Beckman Coulter AMPure XP magnetic beads [65.2% (vol/vol) ratio] and subsequently used for sequencing on a Nanopore minION Mk1B platform using a FLO-MIN114 (R10.4.1) flow cell. The sequencing 1D library was constructed with the SQK-NBD114.24 Native Barcoding Kit 24 V14 (ONT, Oxford, UK) in conformity with the manufacturer’s instructions and long DNA fragment size selection.

Obtained raw reads (2’888’570’766 bp; 802’556 reads; mean read length 3.5 kbp; median read quality 13.7; N50 7.7 kbp) were basecalled with Guppy v6.4.6 (superaccurate basecalling, 400 bps options) prior to quality filtration with chopper v.0.2.0 (4). A minimum quality threshold of Q15 was applied. Additionally, the first 10 bases were removed, and reads <1,000 bps were filtered out (294’536’205 bp; 193’610 reads; mean read length 5.1 kbp; median read quality 16.5; N50 8 kbp), followed by the assembly with Flye 2.9.2-b1786 (5) with the -nano—corr option. The recovered circular chromosome (5’857’139 bp) was classified using GTDB-Tk v2.2.6 (6) and by comparing its 16S rRNA gene (predicted with barrnap 0.9) against the SILVA database (v138.1) (7). Potential genome contamination (completeness 100% and contamination 0.32%) was assessed with CheckM v1.1.3 (8). Coding DNA sequences and tRNAs were predicted by Prokka v1.12 (9) and NCBI’s PGAP pipeline (10). BlastKOALA (11) was used to assign KO identifiers to orthologous genes. Inferences of complete metabolic pathways and general biological functions were conducted with the online KEGG mapping tools using summarized KO numbers. PFAM domains were identified in the proteome using the script pfam_scan.pl with the PFAM database release 32 (12). The growth rate was assessed with the R package gRodon (13). Default parameters were used except where otherwise specified.

The genome was classified as belonging to *Pseudomonas kielensis* by GTDB (99.4% similarity to GCF_026170095.1, 95.81% coverage) and *Pseudomonas kielensis* by SILVA (99.8% similarity to the closest Pseudomonas sequence) databases. It was found to encode 5,362 CDS and 77 tRNAs and to possess 3 rRNA operons. The genome-inferred metabolic reconstructions depicted a copiotroph diderm bacterium with a fast growth rate (estimated duplication time: 1.6 h) and an aerobic heterotrophic lifestyle. Predicted type II and IV secretion systems coupled with associated toxic effectors likely contribute to competition and pathogenicity in a polymicrobial environment.

## Data Availability

All sequence data is available through the National Center for Biotechnology Information (NCBI) via the BioProject PRJNA991734 (Biosample: SAMN36315995, Accession: GCA_030505375.1, SRA: SRR25177532). Annotation files: 10.6084/m9.figshare.24032811.
